# The contribution of personal investment theory of motivation in second language acquisition^☆^

**DOI:** 10.1016/j.heliyon.2023.e16681

**Published:** 2023-05-25

**Authors:** Yanyan Zhang

**Affiliations:** School of Foreign Languages, Sias University, Zhengzhou, 450000, China

**Keywords:** Achievement goals, Facilitative conditions, Motivation theory, Perceived goals of behavior, Personal investment theory, Second language acquisition, Sense of self

## Abstract

As a multi-layered motivation theory, Personal investment theory involves learners in the process of learning, due to multiple components, namely sense of self, facilitative conditions, and perceived goals of behavior. Investment has been described as an essential contributor to the second language learning process along with motivation. It specifies ‘how’ the actual learning occurs in a learning situation in various contexts (e.g., academic and non-academic) both formally and informally. Despite the scarce adoption of Personal investment theory in second language research, one can pose the question of how this theory can contribute to mainstream second language theorizing. This article aims to provide second language researchers with a detailed description of the Personal investment theory. The theory adopts a multi-layered approach to understanding the reasons for learners' investment in a certain domain. The paper presents a general picture of the key principles of Personal investment theory, illuminating the interactions between this theory and research in language education.

## 1 Introduction

Motivation is known as one of the main aspects of human manner, which contributes to the extent to which people devote resources to their goals [1] Accordingly, there has been a growing interest in shedding light on the relationship between this construct and educational achievement in the academic setting [2]. In the same vein, motivation has proved to be essential in achieving success in general, and in a Second Language learning (L2), in particular. However, in recent decades, the description of motivation as a key contributor to the effectiveness of language learning has been criticized [[3], [4], [5]]. A comprehensive understanding of learners' motivation to learn another language requires consideration of these distinct yet interrelated factors. Therefore, all theories of motivation need to consider the variety of factors that can account for the reasons behind the students' desire to invest their time, energy, and even money in a specific domain such as language learning [6].

Undoubtedly, the diversity of theories can enrich theoretical research as researchers are provided with a wider plethora of theoretical concepts, which can be useful in the investigation of the factors contributing to complex motivational issues. Personal investment (PI) theory as a prospective agenda provides a possible schema through which motivation can be understood more deeply in the context of learning L2 [7]. The PI theory seeks to answer the question of what factors drive people to devote various resources including time, talent, and energy to a specific task. This theory is mainly based on the assumption that motivation should not be considered a stable trait of an individual [8]. On the contrary, it maintains that all people have these resources; however, they choose to invest themselves in different domains [6]. Investment is described as one’s commitment to the objectives, procedures, and identities associated with the learning process. These factors are constantly discussed in various communal relations and power constructions [9]. Moreover, investment refers to the establishment of the social and historical relationship between L2 learners and the target language, and their triviality to learn and practice it. Students' investment in L2 learning is encouraged by the role L2 plays in helping them to access an extensive array of representational and material resources. This, in turn, will enhance the importance of their cultural principal and social command [10]. Although a student may be motivated, he/she may, for example, avoid the potential chances to speak in face of some barriers, including a racist, sexist, or homophobic atmosphere in the classroom. During the last decades, identity and investment have come to be viewed as foundational in L2 education [11,12].

As mentioned by Ref. [13], motivation is viewed as a unitary dimension of “personality” which categorizes students based on the dual dimensions of personality (good/bad, impulsive/analytical, motivated/unmotivated, introvert/extrovert, anxious/confident). However, the concept of investment aims to draw attention to how L2 learner is related to the changing social world. Accordingly, it views the L2 learner as an individual possessing a complex identity and multiple desires [14] The notion of investment is also concerned with the student as a person who embarks on learning an L2. This is indicated by complex identities which are both context and time-dependent. Investment involves forming complex social relationships between L2 learners and their unclear desire to put to use (speak) the L2 in the classroom. As a motivation-related theory, PI theory provides the researchers with a relative and non-culture-specific agenda to examine motivation [15,16]. This theory goes beyond the cultures and is based on the assumption that motivation emanates from a sense of self, perceived goals, and facilitative circumstances.

Whether or not a learner invests in the L2 learning process centers on the social interaction and power relations formed in the classroom. Indeed, learners exchange information in a foreign language communicatively. Moreover, they are also involved in constant evaluation of who they are and how they are related to the surrounding world [17]. As a social-cognitive theory, PI theory deals with how people opt for devoting their energy, ability, and time to activities [16,18]. This theory deals with the reasons behind the individuals' desire to spend their energy, time, and money in the process of doing specific activities [19]. Personal investment theory assumes that motivation should not be viewed as a stable trait possessed by an individual [20]. Nevertheless, motivation implies that the motivated person has all resources, including knowledge, patience, and time, among others. These resources drive him/her to opt for investing in different domains [21]. As a multi-layered framework, PI theory enables researchers to figure out several factors contributing to how a learner chooses to spend time and energy learning L2 [6].

Although studies indicate that various groups of learners acknowledge similar scholastic goals and ideals, groups possess different motivational designs. These patterns represent cultural-related motivational scopes. Indeed, PI emphasizes the subjective meaning that individuals make of achievement situations, which is a reflection of their cultural-related belief systems [22]. According to Ref. [23], motivational theories suffer from some shortcomings as they do not take into account the complicated relations between changing identities, the milieu of the classroom, the social environment, and their cultural experiences. Indeed, these issues have not been adequately covered in the psychological theories of L2 learning-related motivation. It is worth mentioning that several types of theories account for the motivations related to the process of L2 learning. However, among these theories, PI theory attaches great importance to the contribution made by social and cultural contexts to shaping motivational patterns associated with learners' performance on achievement tasks.

Even though PI theory enjoys a rather long history in learning psychology and multicultural psychology [7,16,24], its use has not been widespread in the context of L2 motivation. This theory involves a comprehensive analysis and a picture of the reasons for learners' commitment to learning an L2 [25]. This process recognizes an L2 learner as a social individual who takes on an active role when he/she is engaged in the socially interactive practice of L2 learning. Given the above-mentioned points, this study aims to concentrate on just this theory, namely, PI theory, which draws on motivation to demonstrate its important role, especially in enriching L2 motivation research. It also paves the way for the provision of practical recommendations to L2 teachers.

## 2 Review of the literature

### 2.1 Personal investment theory of motivation

In the context of L2, motivation is concerned with the efforts made by the learners to learn an L2, as well as the positive perceptions these learners have of learning the target language and a variety of factors that contribute learning the L2 [5]. In L2 context, motivation is characterized by Ref. [26] as an individual’s willingness to invest many resources to work out an association between one’s commitment to learning an L2 and his/her changing identity Investment involves more than instrumental motivation. This is because such a type of motivation broadly is concerned with a monolithic L2 learner who wishes to get access to material resources that are considered advantages of the target language speakers. This type of motivation has to do with the fixed aspects of one’s personality [14]. A group of learners may feel motivated since they are eager to learn the target language [27]; another group, as mentioned by Ref. [28], may embark on learning L2 since they evaluate themselves as capable of language speakers (self-efficacy); the third group places a high value on gaining proficiency in the target language (value) [29].

The focus of the PI theory is on how a person perceives a situation by making subjective meaning. Such a meaning-construction process helps the researchers to figure out the factors underlying the decision to devote oneself to a particular activity [14]. PI Model which was developed by Maehr has proved to be a comprehensive model of motivation according to which learners' behaviors are the outcomes of their own perceptions of the environment, their academic assignments and activities, the events unfolding around them, and other factors [14].

### 2.2 Components in PI theory

Personal investment theory enjoys several main strengths, such as its holistic multi-layered approach to grasp the real concept of motivation, incorporation of sociocultural impacts, a concentration on influential yet uncovered concepts, and its focus on cross-cultural correspondences and alterations. When it comes to motivation, PI theory adopts a decision-making approach. Based on this perspective, one’s choice to invest oneself in an activity hinges on three core dimensions of meaning [21] which play an essential role in determining PI in specific situations. These components are as follows: Sense of self, facilitating conditions (perceived convenience related to the accomplishment of the goals), and perceived goals (one’s perceptions of goals associated with behavior in given situations) [14].

#### 2.2.1 Sense of self

Sense of self refers to how one perceives oneself concerning one’s feelings and beliefs about oneself. It is concerned with the previous enhancements made at school, level of satisfaction, positive outcomes, and aspirations [30,31]. The sense of self component is defined as the more or less prearranged group of insights, views, and emotional states about who one is [21]. They have highlighted the importance of a sense of self or self-perception in providing a better picture of motivation and learning. The sense of self component embraces the concepts of a sense of competence, Goal-directedness (sense of purpose), Self-reliance, and Social identity. Sense of competence reveals individuals' buoyancy in their capabilities, and the self-evaluation of their position. Goal-directedness is their inclination to set objectives and apply them to shape tasks. As [19] maintained, “purpose in life” may be just one factor in manipulating an individual’s investment of their time and energies. People invest their time, aptitude, and assets to achieve their apparent purpose in life. Self-reliance signifies their capability to continue and flourish by controlling the situation faced. Social identity connotes the interpersonal association persons make with other groups and their recognition of significant other persons [21,32]. All of these insights help individuals' enthusiasm and they act together with motivational goals [33,34]. These aspects were obtained by using the Inventory of School Motivation. The examination of these factors helped to find the differences between learners with various backgrounds. According to Ref. [35], some investigations have paid much attention to future-directed dimensions of the self, turning a blind eye to the motivational outcomes of other self-concepts.

#### 2.2.2 Facilitating conditions

Meaning-making is closely related to environmental conditions, including the social environment where many factors make it easier for an individual to make sense of utterances. The school atmosphere is a significant facilitating condition since learners feel that they belong and that is categorized by a sense of emotional well-being to cultivate learners' education [36]. Some of these factors are more salient than others, influencing one’s ability to extract meaning positively. Facilitating conditions involve various influences, including positive and negative influences of peers, instructors, and family members [14]. Moreover, these conditions can involve the educational setting and the macro socio-cultural types situated in the surrounding environment. Broadly, social environments can either facilitate or deter learners' engagement in learning. A review of the literature reveals a relationship between learners' attitudes toward support provided by parents, instructors, and students and academic achievement [37]. For example, instructors make a precious contribution to raising or lowering L2 learners' motivation (e.g., learners' perceptions of the support provided by the teacher and specific teacher behaviors influence L2 learners' motivation) [[38], [39], [40]]. The majority of these investigations have dealt with the motivational systems used by teachers [39,41]. Investigators have discovered a strong correlation between motivational strategies used by teachers and their learners' motivation associated with second language learning.

Moreover, intuitively, there is a close relationship between positive perceptions of education in an L2 classroom and the availability of a supportive peer group [42,43]. Such a group serves as a key motivating source. This is supported by the evidence provided by the empirical investigations that there is a significant association between peers' perceived motivation in L2 learning and learners' motivation [44]. Additionally, unmotivated peers reduce the learners' motivation [45]. Impressed by the upbeat social climate governing the small groups in the classroom when engaging during L2 learning, students are encouraged to increase their commitment. This, in turn, leads to an increase in motivation [46]. Furthermore, family expectations and the level of support provided by family impact learners' motivation to learn an L2 [47]. Current evidence reveals that parental attitudes influence children’s L2 learning motivation, in addition to the motivation of other age groups (e.g., motivation for entering the university) [48]. Wealthy families are predicted to provide better support and cultivate and improve the setting for their children to learn L2 [49].

#### 2.2.3 Perceived goals of behavior

One more component of PI is learners' perception of goals that this conceptualization has to do with what a person views as failure or success in a situation [50]. This theory implicates cross-cultural elements in the construct of achievement motivation, providing a multi-layered model of motivational goals associated with learning. This theory contrasts with achievement goal theory as the latter has come under question because of its limited conceptualization of goals, as well as its sole focus on performance and mastery goals. This model accounts for three categories of motivational goals, which are deemed important if one intends to work out achievement-related behaviors [34]. These goal categories are performance, social, and mastery goals [51]. Personal investment theory accounts for several goals that can be categorized into mastery, performance, social, and extrinsic ones, all of which have proved to contribute to a better grasp of motivation [21]. Mastery goals have to do with an individual’s intention to enhance one’s competence, while performance goals are concerned with achieving to demonstrate one’s superiority over other peers. Social goals refer to the individual’s endeavor to gain a sense of belonging to a community or a group, and finally, extrinsic goals involve the desire to be recognized socially thanks to his/her accomplishments in school [52]. Perceived goals involve personal incentives, which give a picture of the main motivating features of a task or activity. Personal goals are impacted by the extent to which learners invest in learning. It is also dependent on how failure and success are defined culturally [19]. A study conducted by Ref. [53] has shown that those parents who consider failure as a factor contributing to self-improvement had children with a growth-oriented mindset. In its first version, PI theory dealt with two societal goals, namely, societal connection (the tendency to connect with other learners socially) and societal concern goals (the tendency to help other people) [32]. Recent studies have pinpointed other types of social goals, including the following: social approval goals (the tendency to draw the acknowledgment of parents and teachers), social responsibility goals (the tendency to perform social role obligations), and social status goals (the tendency to raise one’s forthcoming social position through instruction) [54].

The general goal detected by the relevant literature is a task that is impacted by mastery; ego which is impacted by social capability or performance; social unity which involves relational interactions; and extrinsic motivations [55]. There are different relations between goals based on the particular culture [56,57]. For instance, the goals in a culture that emphasizes competition for learning and employment can be mutually supportive. This is evident in the value placed by people in Asian societies on the internalization of both ego and task objectives [56,58].

### 2.3 The contribution of PI theory to L2

Personal Investment approach can also be employed as an integrated framework upon which L2/FL investigators can extend the set of potentially beneficial constructs they rely on. Second or foreign language acquisition is a complicated problem, and investigators are becoming more aware of the different elements that can influence second or foreign language acquisition. For instance Ref. [59], investigated the motivation of Japanese students to learn English. By exploring various aspects of PI approach, they figured out how significant English is to the job-related prospects of Japanese women, who have almost restricted options in the labor market in comparison with men. They also disproved the widespread stereotype that Japanese pupils lack innate desire. The second, which is precisely linked to the previous point, allows PI theory to concentrate on an important element that has received less attention in the literature of second and foreign language acquisition. The influence of assured theoretical paradigms in second or foreign language acquisition motivational investigation can have accidental results by narrowing the investigator's perspective, Because the they concentrate only on the important components postulated in these common L2/FL motivational approaches. For instance Ref. [60],'s social pedagogy model emphasizes the role of integrativeness, and [61]'s model of second language motivational self-system concentrates primarily on the dominance of ought to and the optimal selves.

This theory, in particular, focuses on significant motivational factors that have received insufficient attention in the literature of investigation, comprising parental and peer effects, multiple self-concepts, and social targets [62]. PI approach also prepared us with a viewpoint to investigate and comprehend the overall picture of pupils' progress. In fact, PI approach highlights that learners grow holistically. Learners' performance purposefully according to self-reflection, the search for meaning in life, and awareness of their own strengths and skills in growth. In this approach, what learners do is placed in a greater context that includes key aspects of being and behaving. Pupils become and remain engaged in learning and attainment (personal investment) and are a significant element of their holistic advancing patterns. However, they behave in tandem with a sense of identity and are affected by their social and cultural environment.

## 3 Conclusion and future directions

Undoubtedly, L2 learning is a complex enterprise and it has intrigued L2 researchers over time. Accordingly, they have been trying to examine the role of different factors contributing to L2 learning. L2 learning motivation has been capturing the attention of researchers since decades ago [63]; however, research shows that motivation is a multilayered rather than a monolithic construct; therefore, no theory has fully accounted for its complex nature [47]. The PI theory is a new development in the context of L2 research, providing a new perspective for shedding light on motivation. Investment has proved to be an important concept in L2 classrooms that can accelerate the progress regarding L2 learning [64]. Researchers draw on PI theory as the theoretical framework through which they can spot the variables that play an important role in predicting L2 learners' academic success. This model investigates multiple goals across different cultural settings, offering a more reproductive framework to examine the impact of various types of objectives (e.g., social and achievement) on the quality of learning. Indeed, as pointed out by Ref. [65], PI theory is based on several basic tenets of social cognitive theory. For example, it emphasizes the relationship between learners' motivations and how they perceive the support and value placed on education in their social environment, as well as their convictions regarding themselves as learners.

One can use PI theory as a holistic and multi-layered framework to implicate a wide-ranging set of relevant constructs that L2 researchers can draw upon. Leaners' actions and behaviors are all purposeful and driven by their self-reflection. Indeed, they try to derive a sense of purpose and meaning in life, doing their best to become aware of their strengths and capabilities during their development. PI theory puts the learners' behaviors and actions into a wider context, which includes the core dimensions of being as well as doing. Accordingly, L2 teachers need to consider PI in the classroom as motivation must be backed up by this theory. This review shows that PI theory has come close to accomplishing this goal, thanks to the role it plays in comprehending the difficulty of L2 motivation. These new insights provide educators with strategies to encourage learners to invest in their efforts to acquire L2.

The theory provides an integrated and multidimensional framework to work out a multitude of factors impacting learners' decisions regarding investment in L2 learning. Accordingly, it paints a clear picture of the critical issues that have been marginalized in the L2 literature. A review of the literature reveals that the support provided by family members, instructors, and peers can play a facilitative or prohibitive role in learners' engagement during learning [66].The learners' perceptions of others' supportive measures and caring make a great contribution to their achievement motivation [67]. Indeed, learners can follow several kinds of goals which can significantly impact engagement and achievement in school [68]. This theory brings about multiple advantages when it is compared to other theories of achievement motivation as it has deemed culture as a non-deciding factor in achievement motivation [52]. From the outset, PI theory was meant to be culture-sensitive by incorporating multiple goals deemed important by learners who belong to different cultures [52].

This theory also gives several practical references for L2 teachers and experts on how to motivate learners. Given that L2 teachers have close interaction with L2 learners, they are expected to lay the groundwork for learners' investment in L2 learning so that learners are encouraged to devote themselves to language learning [69]. One way to encourage L2 learners in this regard is the role teachers can take on to place value on the important role of PI and devote a portion of class activities to the learners' investments. PI theory attaches great importance to the significant role of social factors in paving way for conditions that push learners to allocate time and energy or refrain from L2 learning. This theory accounts for three levels of social factors contributing to motivation. First, the category of personal incentives consists of social goals as well the agreed-on task and ego goal considerations. Second, a sense of self involves self-identity which is associated with one’s perception of social or cultural affiliation and its ramifications. Third, perceived freedom for taking part in various activities is often impacted by social aspects, including affiliation, chances to facilitate and/or socialize with others, and family relationships.

As far as L2 learning is concerned, teachers can play an important role in learners' L2 self-concept by providing feedback on their performance. The provision of such feedback on learners' L2 progress would create a perception of one’s competence. This enables the learners to make progress towards gaining a skill, which, in turn, improves self-concept. If instructors let learners know how well they are doing and provide information on the skills and knowledge they have acquired, learners can gain a more positive self-concept.

Given the important role played by parents in forming children’s enthusiasm to learn an L2 in the literature [49], parenting packages are of paramount importance since they are aimed at encouraging their involvement, modifying ineffective parental behavior, and strengthening advocating parenting style that reinforces intrinsic motivation in L2 learning. A positive self-concept is associated with better performance [70].

Personal investment theory influences three facets of learners' performance: first, the direction taken by learners, which refers to the decisions they make regarding the allocation of their attention for finishing tasks, second, students' perseverance, which has to do with their desire to deal with instructional activities for longer periods, and third, learners' enduring motivation, which refers to their desire to modify explicit activities in succeeding lessons. In the same vein [9], asserted that learners' verbal, educational, cultural, and individual capital pave the way for enhanced learning in the L2 classroom.

More empirical research should be carried out in which their focus will be on L2 learners' investment in-class activities that can enhance L2 motivation and proficiency among students. Another strand of research can focus on examining what opportunities should be provided to L2 learners so that they can invest more in learning across time and place. Correspondingly, more studies should be done on how learners with a low level of investment can take advantage of innovative approaches to L2 learning. Similarly, more research could be carried out to regulate the role of this theory in other cultural perspectives since this theory offers enhanced alternating issues in reviewing motivational objectives in various cultural situations.

## Author contribution statement

All authors listed have significantly contributed to the development and the writing of this article.

## Data availability statement

No data was used for the research described in the article.

## Additional information

No additional information is available for this paper.

## Declaration of competing interest

The authors declare that they have no known competing financial interests or personal relationships that could have appeared to influence the work reported in this paper
